# Bevacizumab-Irinotecan combination therapy in recurrent low-grade glioma, previously treated with chemo-radiotherapy: a case report

**DOI:** 10.3389/fonc.2023.1244628

**Published:** 2023-09-20

**Authors:** Barbara Castelli, Carla Fonte, Milena Guidi, Marco Tellini, Marco Di Nicola, Alessandro Iacono, Anna Maria Buccoliero, Daniela Greto, Lorenzo Genitori, Iacopo Sardi

**Affiliations:** ^1^ Health Sciences Department, University of Florence, Florence, Italy; ^2^ Neuro-Oncology Unit, Meyer Children’s Hospital IRCCS, Florence, Italy; ^3^ Radiology Unit, Meyer Children’s Hospital IRCCS, Florence, Italy; ^4^ Pathology Unit, Meyer Children’s Hospital IRCCS, Florence, Italy; ^5^ Radiotherapy Unit, University of Florence, Florence, Italy; ^6^ Neurosurgery Unit, Meyer Children’s Hospital IRCCS, Florence, Italy

**Keywords:** low-grade gliomas, bevacizumab, irinotecan, pilocytic astrocytoma, case report

## Abstract

Low grade gliomas (LGGs) of pineal region are usually difficult to remove and they frequently relapse or progress after front line chemotherapy. Bevacizumab-Irinotecan (BEVIRI) combination has been successfully attempted in children with recurrent LGGs, in most cases not previously irradiated. The efficacy of bevacizumab has also been described in radiation necrosis. Considering the possible overlapping of radiation treatment effect and disease progression and difficulty in differentiating, we report on the use of BEVIRI in a case of a recurrent relapsing low-grade glioma of the pineal region, subjected to multiple neurosurgical interventions, also treated with a carboplatin-etoposide regimen and a radiation course, at present at one-year follow-up showing a stable response, with no adverse events.

## 1 Introduction

Low grade gliomas (LGGs) are the most common pediatric brain tumors (1), accounting for one third of all primary central nervous system (CNS) tumors in children less than 18 years of age (2).

Prognosis is excellent when complete resection is obtained (2). However, frequently, resection is not feasible because of their location (2).

Chemotherapy has been considered for young children with incompletely resected pediatric LGGs, to delay radiotherapy, and for recurrent tumors in unfavorable locations as hypothalamic or pineal gliomas (3). Multiple chemotherapeutic regimens have been employed in children with LGGs (1). However, at least half of children with LGGs treated with front line chemotherapy experience a relapse/progression and may therefore need a second-line chemotherapy (2). Recent advances in understanding molecular characteristics of pediatric LGGs have affected classification and treatment approaches (3). Various agents that target the MAPK pathway, such as MEK, BRAF, mTOR inhibitors, are currently attempted in pediatric LGGs with encouraging results (3).

Considering standard chemotherapy regimen, Bevacizumab-based therapies, including bevacizumab (a humanized murine monoclonal antibody against vascular endothelial growth factor, VEGF) plus irinotecan (campothecin derivate that inhibits the nuclear enzyme, topoisomerase I), have been used successfully in children with LGGs (4). Bevacizumab may have a direct role on vascular normalization, improving delivery of cytotoxic drugs (2) and decreasing interstitial pressure (2). Irinotecan may simultaneously potentiate the action of bevacizumab by inhibiting hypoxia-inducible factor 1 (2). However, only a few patients have been treated with these regimens after having also received brain irradiation. VEGF inhibition has been proposed as a potential strategy to combat cerebral radiation necrosis (5), reducing neurological symptoms and improving quality of life (6).

Herein we reported a case of a recurrent pineal low-grade glioma, relapsed after chemotherapy, radiotherapy, and surgical resections, showing response to bevacizumab-irinotecan regimen as rescue therapy.

## 2 Case report

**Figure 1:**
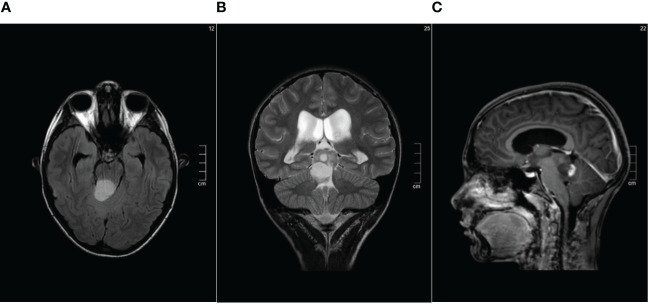
Pre-surgical brain MRI performed in July 2013 showing the solid-cystic lesion of the pineal region **(A)** Axial FLAIR sequence. **(B)** Coronal T2-weighted sequence. **(C)** Contrast enhanced Sagittal T1- weighted sequence.

In May 2013, a 15-year-old male was admitted to our hospital for headache, vomiting and diplopia. Imaging revealed a solid-cystic lesion of the pineal region, causing aqueductal stenosis. He underwent an endoscopic third ventriculostomy, and in July 2013 craniotomy removal of the lesion (**Figure 1**). The histopathological examination revealed Pilocytic Astrocytoma (PA) (WHO-grade I).

**Figure 2:**
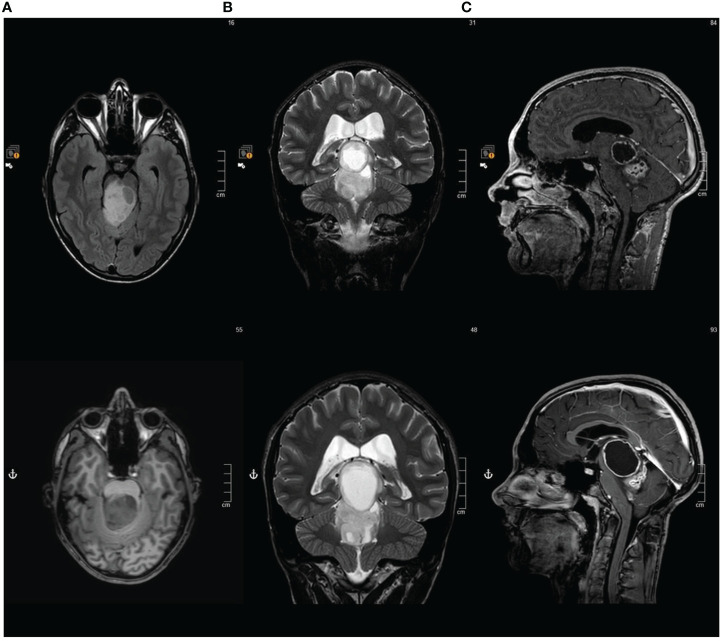
Brain MRI at first and second disease progression **(A)** Axial FLAIR sequence. **(B)** Coronal T2- weighted sequence. **(C)** Contrast enhanced Sagittal T1-weighted sequence.

At three-year follow-up magnetic resonance imaging (MRI) of the brain showed disease progression (**Figure 2**), so the patient was started on a lower-dose chemotherapy regimen modified by the Milan strategy (7) consisting of carboplatin (400 mg/m2/die, day 1) and etoposide (100 mg/m2/die, day 1-3) every four-six weeks from November 2016 to June 2017, when the lesion expanded (**Figure 2**). In June 2017 the patient underwent a further neurosurgery. Moreover, from September to October 2017 a radiation volumetric modulated course, for a total dose of 50,4 Gy in 28 fractions of 1,8 Gy/day was delivered, without complications.

**Figure 3:**
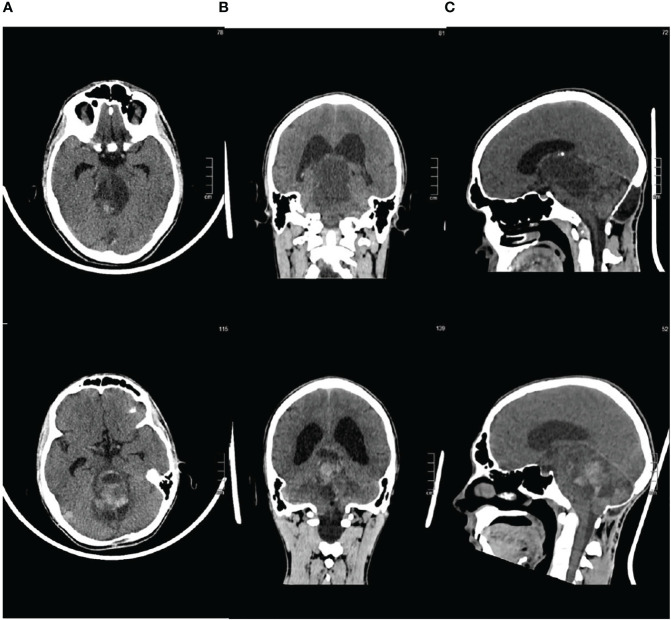
Brain CT scan at third and fourth disease progression **(A)** Axial sequence. **(B)** Coronal sequence. **(C)** Sagittal sequence.

In February 2019, for the appearance of neurological symptoms (progressive blunting of sensorium), an MRI of the brain was urgently requested, demonstrating an increase of the solid-cystic lesion requiring neurosurgical intervention (**Figure 3**).

In December 2019, the patient complained of deterioration of motor coordination with walking difficulties. An MRI of the brain again revealed a disease progression with an increase in size encompassing the fourth ventricle and invading the third one (**Figure 3**). The patient underwent a partial total removal, and his postoperative course was without complications. The histopathological examination confirmed a Pilocytic Astrocytoma, GFAP +, p53 non over expressed, non IDH-mutated, with a Ki-67 up to 4-5% (**Supplementary Material 1**). Therefore, in January 2020 it was multidisciplinary decided to start the patient on a third-line treatment (BEVIRI) consisting of intravenous Bevacizumab 10 mg/kg and Irinotecan 125 mg/m2 every four weeks for twenty-four cycles. Serial MRI showed stable findings, with a minimal reduction in the size of the known pineal remnant. Occasionally, at arterial blood measurements before bevacizumab administration, systolic hypertension (CTCAE grade 1) was found, without ever having the need of antihypertensive medications or therapy adjustment. No further adverse events have been reported, in particular no proteinuria, no bleedings and no hematological alterations. The course was overall well tolerated, in absence of complications. The last cycle was administered in December 2021.

**Figure 4:**
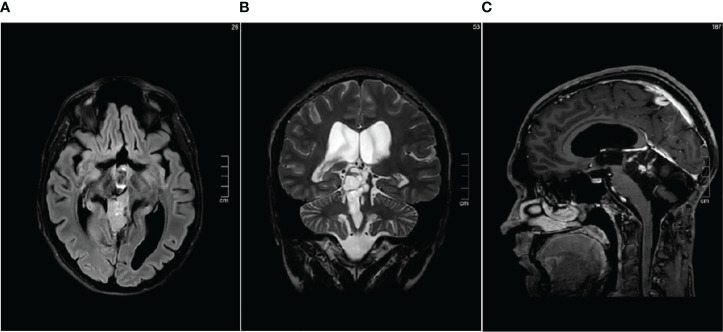
Brain MRI performed in January 2023 showing stable disease **(A)** Axial FLAIR sequence. **(B)** Coronal T2-weighted sequence. **(C)** Contrast enhanced Sagittal T1-weighted sequence.

At most recent follow-up (January 2023, a follow-up time of 12 months from the last BEVIRI dose) imaging has remained stable over and the child has had no symptomatic recurrence (**Figure 4**).

## 3 Discussion

We described the case of a recurrent relapsing low-grade glioma of the pineal gland, subjected to multiple neurosurgical interventions, also treated with a carboplatin-etoposide regimen and at recurrence with radiotherapy. Bevacizumab-irinotecan combination was started as rescue therapy for predominantly cystic pseudoprogression after radiotherapy.

Management of LGGs can be a challenge, particularly when refractory or recurrent following standard treatments (8). Recent studies have reported the results of the use of bevacizumab with or without irinotecan as treatment for children with progressive/recurrent LGGs with at least temporary efficacy in a significant percentage of patients (1).

De Marcellus et al. reported that Bevacizumab-Irinotecan was highly effective in pediatric recurrent LGGs who have failed standard chemotherapy regimens whatever their clinical characteristics (2). They analyzed 72 patients with recurrent LGGs (but only two patients previously treated with radiotherapy) (2).

Packer and colleagues described the effectiveness of bevacizumab and irinotecan in a cohort of 10 children with multiple recurrent LGGs (9 patients had progressed after three or greater chemotherapy regimens and one had also received radiation therapy) (9).

Carefulness must be paid whereas combination therapies of radiation and cytotoxic drugs are often related to potential acute or delayed toxicities, including neurotoxicities (10). Previous chemotherapy, particularly bevacizumab, is common in cancer patients with Posterior Reversible Encephalopathy Syndrome (PRES) (11).

Gorsi et al. demonstrated that single-agent bevacizumab is efficacious in the management of recurrent or refractory pediatric LGGs, with radiographic and clinical responses similar to those described for bevacizumab- based therapies (4). They reported on two patients with Juvenile pilocytic astrocytoma (JPA) started on bevacizumab: one patient, previously exposed to chemo-radiotherapy had no response; the other patient, previously exposed to radiation, responded but showed radiographic progression after discontinuation, so he received a second course with partial response (4).

Hwang and colleagues tested the hypothesis of repeated bevacizumab courses in pediatric LGGs, affecting long term-control and showing favorable efficacy on subsequent rechallenge (12).

Previous studies on the combination therapy focused on children with progressive/recurrent LGGs who had not received radiotherapy (1).

A familiar challenge for neuroradiologists and neuro-oncologists is differentiating between radiation treatment effect and disease progression in the CNS (13). In fact both entities are often characterized by an increase in contrast enhancement on MRI and may occur either in close temporal proximity to the treatment or later in the disease course (13).

Radiation necrosis, typically presenting 3 to 12 months after radiotherapy but also years after treatment (5), can result in worsening neurologic symptoms (14). Vascular damage followed by VEGF expression at high levels is a key mechanism for radiation brain necrosis development (6). The treatment for radiation necrosis typically consists of steroids, which nonspecifically reduces edema, but is associated with numerous significant side effects (14).

Several studies have established that bevacizumab is an effective treatment for radiation brain necrosis. Bevacizumab, inhibiting VEGF, can reduce radiation necrosis by decreasing capillary leakage and the associated brain edema (15).

Levin et al. conducted a a placebo-controlled double-blind study, showing the Class I evidence of bevacizumab efficacy in the treatment of central nervous system radiation necrosis (16).

Foster et al. reported on the successful use of bevacizumab also for the treatment of radiation-induced tumor enlargement in five symptomatic children with LGGs (1).

Children who do not respond to bevacizumab therapy may have progressive disease rather than radiation necrosis, suggesting both a therapeutic and diagnostic role of bevacizumab in radiation necrosis (14). Liu et al. described four children with pontine gliomas who received bevacizumab as a treatment for the radiation necrosis: three children had significant clinical improvement allowing interruption of steroid use, instead one child continued to decline (14). In retrospect, this patient had disease progression, not radiation necrosis (14).

Recurrence of radiation necrosis after temporary improvement by bevacizumab treatment has been described, controlled by bevacizumab rechallenge (17). Jeyaretna et al. described the case of a 35-year-old man with a grade 2 irradiated oligoastrocytoma, who presented initially significant improvement then a worsening of his condition after bevacizumab treatment (5). They hypothesized that the phenomenon of over-pruning, caused by bevacizumab treatment, is predicted to lead to vascular insufficiency, which can exacerbate hypoxia and necrosis (5).

In our case the absence of recurrence after 12 months from the suspension of the treatment leans towards disease progression, rather than radio necrosis. However, both components may have contributed to determine the clinical and radiological relapsing picture.

In conclusion, our report supports that Bevacizumab-Irinotecan treatment should be evaluated as rescue therapy regimen in patients with relapsing or refractory LGGs after exposure to standard treatments inclusive of radiotherapy, taking into account the risk-benefit ratio.

This is a limited study, reporting our favorable experience only in a single patient in a 12-month follow-up. Further studies are expected to verify sustained response and more data on larger cohorts are needed before any conclusions should be drawn.

## Timeline


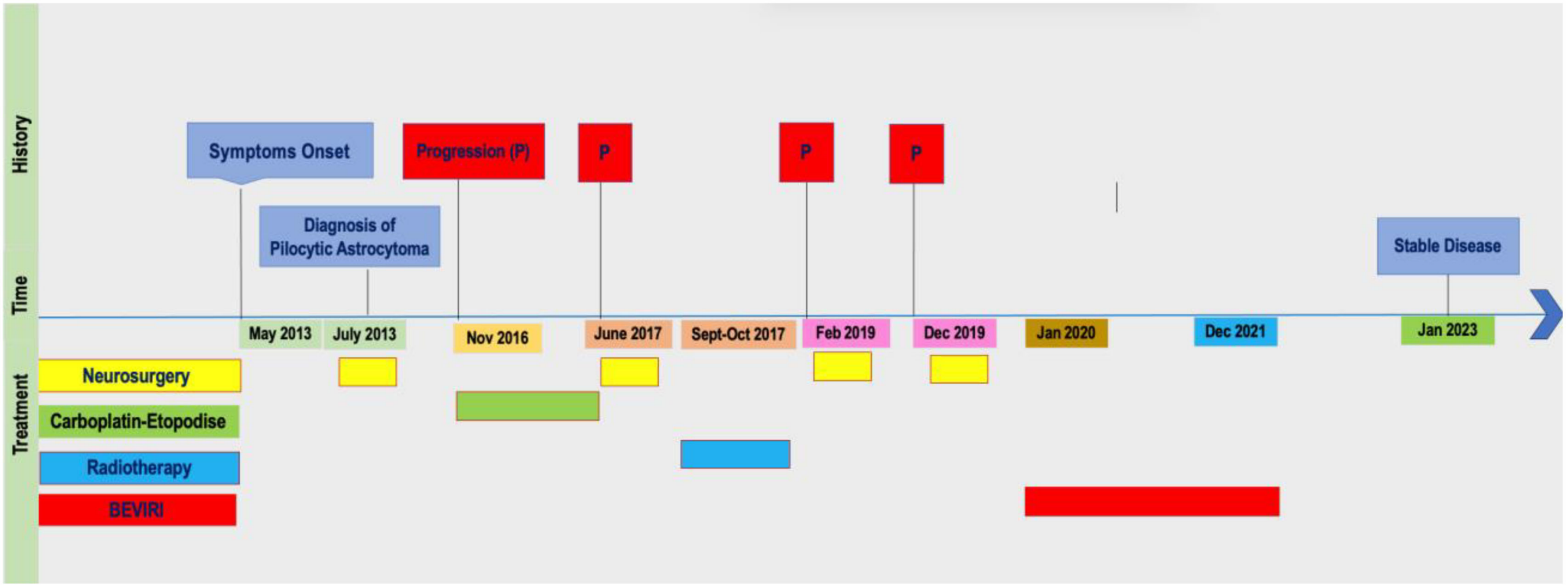


## Data availability statement

The original contributions presented in the study are included in the article/**Supplementary Material**. Further inquiries can be directed to the corresponding author.

## Ethics statement

Written informed consent was obtained from the individual(s) for the publication of any potentially identifiable images or data included in this article.

## Author contributions

All authors contributed to the article and approved the submitted version. BC and IS composed the manuscript, CF, MG, MT, MD, DG, LG revised it critically, AI provided figures, AB provided histological analysis. All authors contributed to the article and approved the submitted version.
