# Corrigendum: Bevacizumab-Irinotecan combination therapy in recurrent low-grade glioma, previously treated with chemo-radiotherapy: a case report

**DOI:** 10.3389/fonc.2024.1372295

**Published:** 2024-02-01

**Authors:** Barbara Castelli, Carla Fonte, Milena Guidi, Marco Tellini, Marco Di Nicola, Alessandro Iacono, Anna Maria Buccoliero, Daniela Greto, Lorenzo Genitori, Iacopo Sardi

**Affiliations:** ^1^ Health Sciences Department, University of Florence, Florence, Italy; ^2^ Neuro-Oncology Unit, Meyer Children’s Hospital IRCCS, Florence, Italy; ^3^ Radiology Unit, Meyer Children’s Hospital IRCCS, Florence, Italy; ^4^ Pathology Unit, Meyer Children’s Hospital IRCCS, Florence, Italy; ^5^ Radiotherapy Unit, University of Florence, Florence, Italy; ^6^ Neurosurgery Unit, Meyer Children’s Hospital IRCCS, Florence, Italy

**Keywords:** low-grade gliomas, bevacizumab, irinotecan, pilocytic astrocytoma, case report

In the published article, there was an error in affiliations **2,3,4,6**. Instead of “**Meyer Children’s Hospital Scientific Institute for Research, Hospitalization and Healthcare (IRCCS)**,” it should be “**Meyer Children’s Hospital IRCCS**.”

The authors apologize for this error and state that this does not change the scientific conclusions of the article in any way. The original article has been updated.

