# Supplementary material for: Bevacizumab-Irinotecan combination therapy in recurrent low-grade glioma, previously treated with chemo-radiotherapy: a case report
Source: Front Oncol. 2023 Sep 20;13:1244628. doi: 10.3389/fonc.2023.1244628 (PMC10547897; doi:10.3389/fonc.2023.1244628)
Supplement: Supplementary file 1 [file Presentation_1.pptx]

## Slide 1
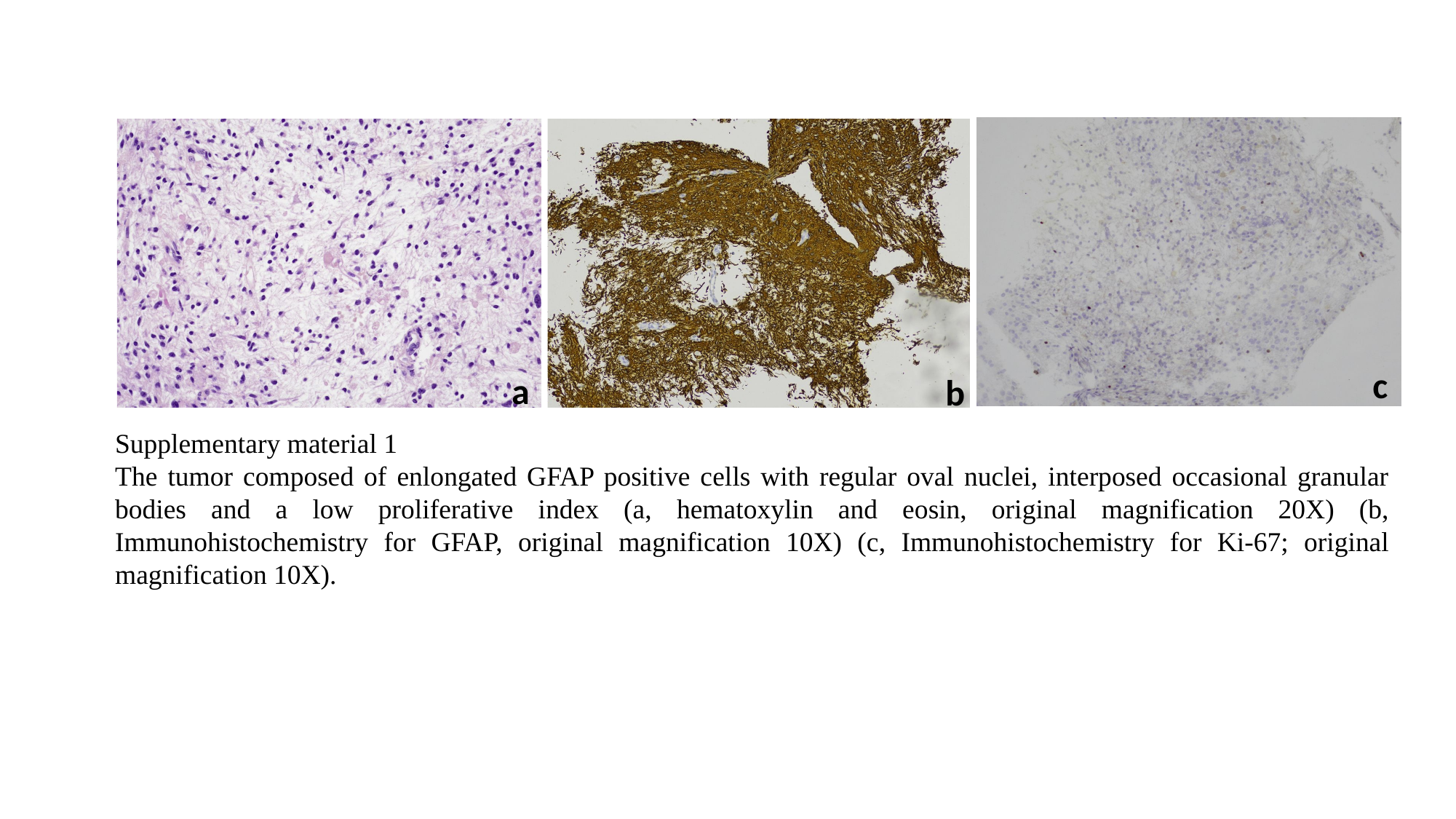

c
a
b
Supplementary material 1
The tumor composed of enlongated GFAP positive cells with regular oval nuclei, interposed occasional granular bodies and a low proliferative index (a, hematoxylin and eosin, original magnification 20X) (b, Immunohistochemistry for GFAP, original magnification 10X) (c, Immunohistochemistry for Ki-67; original magnification 10X).
